# Association between Obesity and Atrial Function in Patients with Non-Valvular Atrial Fibrillation: An Echocardiographic Study

**DOI:** 10.3390/jcm13102895

**Published:** 2024-05-14

**Authors:** Martina Pucci, Vittoria Gammaldi, Luca Maria Capece, Daniele Paoletta, Adelaide Iervolino, Mariateresa Pontoriero, Marina Iacono, Pasquale Megaro, Roberta Esposito

**Affiliations:** Department of Clinical Medicine and Surgery, Federico II University Hospital, 80131 Naples, Italy; martina.pucci@unina.it (M.P.); lucamaria.capece2@unina.it (L.M.C.); d.paoletta@studenti.unina.it (D.P.);

**Keywords:** non-valvular atrial fibrillation, obesity, left atrial strain, speckle-tracking echocardiography (STE)

## Abstract

**Background:** Obesity is a public health problem which prevalence has increased worldwide and is associated with different degrees of hemodynamic alterations and structural cardiac changes. The aim of the study is to investigate the impact of body mass index (BMI) on left atrial function using standard and advanced echocardiography in a population of patients with non-valvular atrial fibrillation (AF). **Methods:** 395 adult patients suffering from non-valvular AF, divided into three tertiles based on BMI value, carry out a cardiological examination with standard and advanced echocardiography. **Results**: Peak atrial longitudinal strain (PALS), a measure of left atrial function, is lower in the tertile with highest BMI (14.3 ± 8.2%) compared to both the first (19 ± 11.5%) and the second tertile (17.7 ± 10.6%) in a statistically significant manner (*p* < 0.002). Furthermore, BMI is significantly associated independent with the PALS by multilinear regression analysis, even after correction of the data for CHA_2_DS_2_-VASc score, left ventricular mass index, left ventricular ejection fraction, E/E’ ratio and systolic pulmonary arterial pressure (coefficient standardized β = −0.127, *p* < 0.02; Cumulative R^2^ = 0.41, SEE = 0.8%, *p* < 0.0001). **Conclusions:** BMI could be considered an additional factor in assessing cardiovascular risk in patients with non-valvular atrial fibrillation, in addition to the well-known CHA_2_DS_2_-VASc score.

## 1. Introduction

Obesity represents a serious and constantly growing public health problem [1]. In recent decades, the prevalence of obesity has increased worldwide [2]; the average BMI (Body Mass Index) has grown globally, both in men and women [3]. The increase in body weight is due to a positive energy balance; however despite studies suggesting that the main cause of obesity is an abnormal caloric intake [4], it must be considered that there are also behavioral factors, which have recently acquired greater importance, such as the spread of increasingly sedentary behaviors and work activities [5]. Furthermore, it must be considered that the development of obesity is also associated with socio-economic status, as well as environmental factors, which can determine epigenetic modifications through gene-environment interaction [6]. In obese subjects, adipose tissue dysfunction occurs, causing a pro-inflammatory state and an imbalance in the production of adipokines, which are associated with the development of cardiovascular diseases (CVD), as well as some types of cancer [7]. Furthermore numerous recent evidence suggested a role of epicardial adipose tissue in the development of atrial fibrillation, in fact several potential arrhythmogenic mechanisms have been attributed to epicardial adipose tissue, including myocardial inflammation, fibrosis, oxidative stress, and fat infiltration. Collagen deposition and subsequent fibrosis may be the substrate for atrial fibrillation [8]. Altered myocardial metabolism may be one of the potential mechanisms underlying obesity-related heart disease. Obesity, in fact, causes an increase in the oxidation of fatty acids and a reduction in the oxidation of glucose [9]. These metabolic alterations modify cardiac metabolism, resulting in an alteration of the efficiency of the cardiac pump and reduced energy production with increased incidence of heart failure [10]. The various classes of obesity are associated with different degrees of hemodynamic alterations and structural changes [11]. The ejection fraction of the left ventricle, an expression of systolic function, is usually preserved in obese subjects. However, new sensitive measures of myocardial function, such as strain imaging in echocardiography and magnetic resonance imaging, have allowed the detection of subclinical alterations even in the presence of a preserved left ventricular ejection fraction [12]. The prevalence of altered myocardial strain in obese patients, varies from 37% to 54%. Subclinical functional abnormalities of the right ventricle may also be present [13]. It has also been observed that higher values of left ventricular mass are found in obese subjects, as well as high diastolic filling pressures in class II and III obese subjects [14]. Prevalence of diastolic dysfunction in obese patients varies from 23% to 75%, depending on the diagnostic criteria used [15]. BMI appears to be a significant predictor of left atrial size [16]. It has been demonstrated that left atrial dilatation is present in obese patients, even when they do not have other cardiovascular diseases. The importance of accurate evaluation of the size of the left atrium in obese patients was also underlined in a recent paper that analyzed the difference between indexing the atrial volume for height or for body surface area in patients with moderate and severe obesity in order to identify the reduction in atrial function assessed with speckle-tracking echocardiography [17]. In one study it was demonstrated that obese and hypertensive patients have a greater risk of having an increased atrial volume compared to normal weight hypertensive patients, indicating that obesity may be a predictive factor of increased atrial size, both individually and in presence of arterial hypertension of which it amplifies the effect [18,19]. A large study of 5282 patients over a follow-up of 13.7 years showed that there was a 52% increase in the risk of new onset atrial fibrillation due to obesity [20]. A meta-analysis demonstrated a 29% increased risk of atrial fibrillation incidence for every 5 unit increase in body mass index (BMI) [21]. In a recent study it was demonstrated that in patients with heart failure with preserved ejection fraction (HFpEF) abdominal obesity was associated with increased incidence of atrial fibrillation, in particular AF incidence rose by 18% per centimeter in circumference [22]. Obesity increases the thromboembolic risk, probably due to the chronic inflammatory state which in itself activates the thrombotic process and plays a role in the pathogenesis of “atrial myopathy” responsible for both HFpEF and AF. Unfortunately, the thromboembolic risk in obese patients is often undertreated, which makes the evaluation of this type of patient highly relevant to allow for more accurate risk stratification and consequently adequate treatment [23].

### 1.1. Role of Echocardiography in Atrial Fibrillation

Echocardiography, together with the medical history, physical examination, electrocardiogram and evaluation of some laboratory parameters, is an examination indicated in all patients with atrial fibrillation (AF). While in valvular forms of AF the thromboembolic risk is high by definition, in non-valvular forms it is estimated through a score known as CHA_2_DS_2_-VASc score.

Echocardiography is useful for assessing thromboembolic risk in patients with atrial fibrillation (AF) as it contributes to the estimation of CHA_2_DS_2_-VASc score regarding the evaluation of congestive heart failure, which is responsible for the letter C in the acronym CHA_2_DS_2_-VASc. It is in fact considered a risk factor chronic systolic heart failure with ejection fraction ≤40% or any form of acute heart failure, both systolic and diastolic (i.e., with preserved ejection fraction). It is clear that, in each of these cases, echocardiography is decisive, being the technique generally used first in the clinical setting to establish the ejection fraction of the left ventricle and for the evaluation of diastolic function.

Many believe that the contribution of echocardiography to the estimation of thromboembolic risk in patients with AF is limited to the evaluation of the ejection fraction. On closer inspection, however, this method also provides further information in the evaluation of vascular disease, which constitutes another significant risk factor for CHA_2_DS_2_-VASc score. Vascular disease includes three entities: previous myocardial infarction, aortic plaques and peripheral arterial disease. In the first two cases, echocardiography is able to make a significant contribution.

As regards previous myocardial infarction, it should first of all be remembered that the standard ECG has a low sensitivity in identifying a stabilized or healed myocardial infarction, whether subendocardial or transmural, especially when it is located in an inferior, posterior or lateral location [24]. Cardiac imaging methods, and among these echocardiography, are particularly useful in revealing the presence of structural and/or functional alterations compatible with a previous myocardial infarction and therefore can allow the diagnosis even when the ECG and clinical history are silent.

As regards aortic plaques, it should be remembered that transthoracic echocardiography allows, in a high percentage of patients, the study of the aortic root and arch and, in some cases, also of the descending thoracic and abdominal aorta Therefore it is possible to use this method to detect the presence of aortic plaques, even complex ones.

Ultimately, therefore, it is clear that echocardiography can contribute to the estimation of thromboembolic risk in the context of CHA_2_DS_2_-VASc score both for estimating the ejection fraction and for the recognition of a vascular, coronary or aortic disease, although the performance of dedicated tests such as color Doppler ultrasound of the supra-aortic trunks, upper and lower limbs are necessary for the evaluation of vascular disease.

### 1.2. Purpose of the Study

The aim of our study was to investigate the impact of BMI on left atrial function using standard and advanced echocardiography in a population of patients with non-valvular AF.

## 2. Materials and Methods

### 2.1. Study Population

The study performed is an observational, non-prospective and monocentric study. We enrolled 395 adult patients suffering from non-valvular AF belonging to our EchoLab at AOU Federico II to carry out a cardiological examination and echocardiographic evaluation (NeAfib-echo registry).

Regarding the clinical characteristics of the enrolled patients, exclusion criteria were considered: known coronary artery disease, known cardiomyopathies, significant valvular diseases, previous cerebral ischemic episodes and known oncological pathologies.

At baseline anthropometric parameters were recorded for each patient; systolic and diastolic blood pressure values and heart rate were measured at the beginning of each echocardiographic exam; for each patient the BMI and the CHA_2_DS_2_-VASc score were obtained.

### 2.2. Echocardiography

Echo Doppler exams were performed by a Vivid E95 ultrasound machine (Horten, Norway) equipped with a 2.5 MHz phased-array transducer according to the American Society of Echocardiography (ASE)/European association of Cardiovascular Imaging (EACVI) standardization [25].

Evaluation of the ejection fraction (EF) of the left ventricle, the left ventricular mass indexed to height powered to 2.7 (LVMi), the global longitudinal strain (GLS) of the left ventricle, the diastolic function by Doppler evaluation of the transmitral pattern and tissue Doppler of the septal and lateral mitral annulus was carried out. Particular attention was paid to the quantification of left atrial dimensions and function. All echocardiographic measurements were performed on an average of three consecutive cardiac cycles of good quality, at high frame rate (40–80 frames/s).

#### 2.2.1. Atrial Dimension

Atrial dimensions are measured at the end of ventricular systole, when the chamber is at its largest. The methods based on the measurement of left atrial (LA) size in an anteroposterior linear dimension by M-mode or 2D echocardiography in parasternal long-axis view don’t represent the true LA size, particularly when LA dilatation is present, resulting in an asymmetric shape of the LA. For this reason, LA-indexed volumes should be assessed for quantification of LA size [26,27].

**Figure 1:**
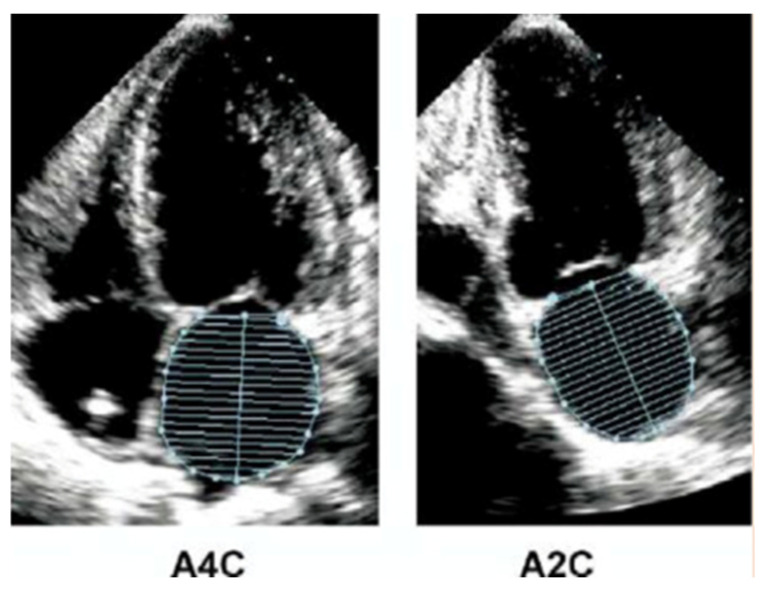
Modified Simpson method (biplane disk summatio) for quantification of left atrial volume index.

In our study we performed the modified Simpson method (biplane disk summatio): the atrial volume is calculated by adding the volume of a stack of cylinders of height “h” and with the area which is calculated from the orthogonal transverse axes (Figure 1). The size of the left atrium depends on gender. However, gender differences are still taken into account when indexing values for body size [28]. The most recommended indexing method is by body surface area (BSA) [29]. Currently the upper limit of left atrial volume indexed for BSA is set at a value of 34 mL/m^2^ [30].

#### 2.2.2. Estimation of Left Atrial Function by Speckle Tracking Echocardiography

**Figure 2:**
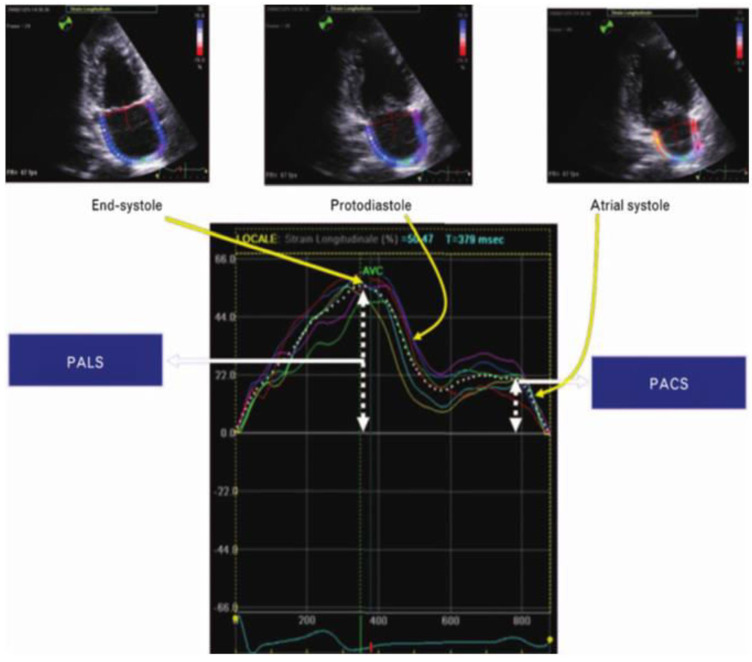
Evaluation of left atrial function using longitudinal strain: peak atrial longitudinal strain (PALS) and peak atrial contraction strain (PACS).

The left atrium modulates left ventricular filling through its functions as reservoir, conduit and pump. During ventricular systole and isovolumetric relaxation, when the atrioventricular (AV) valves are closed, the atrial chambers function as distensible reservoirs that receive blood flow from the venous circulation. The reservoir function depends on the starting atrial volume (end-diastolic), on the relaxation of the atrial fibres, on the distensibility or compliance of the atrial walls and on the descent of the atrio-ventricular plane during ventricular systole [31]. Normally, the considerable capacitance of the left atrium makes it capable of receiving and withstanding increases in venous return which are then transferred to the ventricle through the functions of conductance and contractility. Therefore, the reservoir function of the left atrium-pulmonary vein system may contribute, together with the Frank-Starling mechanism at the level of the underlying ventricle, to the adaptation of the left sections to sudden changes in the flow rate of the left ventricle, which would otherwise lead to the development of stasis and pulmonary congestion. It is important to recognize the interaction between these atrial functions and ventricular performance throughout the cardiac cycle. For example, although the reservoir function is mainly determined by atrial compliance during ventricular systole (and, less obviously, by atrial contractility and relaxation), it is also influenced by the descent of the base of the left ventricle during systole and from the end-systolic volume [32]. The conduit function is influenced by atrial compliance and is reciprocally linked to the reservoir function, but is necessarily related to the relaxation and compliance of the left ventricle. Finally, the atrial pump function reflects the intensity and duration of atrial contractility, but it also depends on venous return (atrial preload), as well as on the end-diastolic pressures of the left ventricle (atrial afterload) and its systolic reserve. In this context, the measurement of atrial dimensions, better defined by volume rather than by atrial diameters, can provide useful elements for obtaining information on the value of atrial pressures and for evaluating the diastolic function of the left ventricle. An echocardiographic method for the evaluation of left atrial function is speckle tracking echocardiography (STE), a system of observation of the “speckles” during the cardiac cycle: speckles are a set of pixels (dots) that form the standard 2D grayscale echocardiographic image [33]. Speckles can be recognized in an entire region and followed throughout the cardiac cycle. This is possible by recording 2D images with a temporal resolution of approximately 40–80 frames per second (fps) [34]. The principle is that if two consecutive frames are temporally close, the small variation in the position of the speckle can be easily recognized by the software which in this way manages to encode and follow the deformation of the selected myocardial segments during the entire cardiac cycle using approaches of probabilistic. The system’s computing power allows this operation to be performed for dozens of regions simultaneously along the profile of a 2D image. After acquiring left atrial images in the apical 4 chambers and 2 chambers, a region of interest (ROI) is identified which draws 6 different segments. To trace the ROI at the auricle and the outlet of the pulmonary veins, the direction between the endocardial and epicardial surfaces is extrapolated by the software. We therefore obtain 12 total segments with 12 corresponding curves for which the software then generates an average curve [35]. Two parameters of longitudinal strain of the left atrium are recognized:

- *Peak atrial longitudinal strain (PALS)*, measured at the end of the atrial reservoir phase. Which in normal subjects is greater than 40%. In patients with atrial fibrillation it has been seen that the reduction in strain reflects structural alterations of the left atrium (wall fibrosis) [36] (Figure 2)
- *Peak atrial contraction strain (PACS)* identified just before the onset of the active phase of atrial contraction [37], which indicates the contribution of the active contraction of the left atrium to the filling phase of the left ventricle [38] and which is lacking in patients with permanent atrial fibrillation.

### 2.3. CHA_2_DS_2_-VASc Score Assessment

Regarding the assessment of thromboembolic risk of patients enrolled in the study, the CHA_2_DS_2_-VASc score was used according to the 2014 AHA/ACC/HRS guideline for the management of patients with atrial fibrillation [39]. In detail, one point was assigned in the case of congestive heart failure or left ventricular dysfunction, one point in the case of arterial hypertension, one point in the case of diabetes mellitus, 2 points in the case of a positive history of stroke, transient ischemic attack (TIA) or thromboembolism, 1 point in case of vascular pathology, 1 point in case of age between 65 and 74 years, 2 points in case of age greater than or equal to 75 years, 1 point in case of female sex.

### 2.4. Statistical Analysis

Statistical analysis was performed by SPSS package, release 12 (SPSS Inc., Chicago, IL, USA). Data are expressed as mean value ± standard deviation (SD) for normal continuous variables; categorical variants are expressed as *n* of patients (%). Continuous variables were compared with the Student *t*-test. Discrete variables were compared with Chi-square (χ2) statistics or Fisher’s exact test when appropriate. Multilinear regression analysis was also performed, including all the variables found to be statistically significantly associated with PALS, adjusting these data for the CHA_2_DS_2_-VASc score. Quantitative data were checked for normality of distribution by Shapiro-Wilk and Kolmogorov-Smirnov tests with a *p*-value < 0.001. Therefore considering that the data do not follow a normal distribution, a non-parametric Kruskal-Wallis test was performed.

The null hypothesis was rejected at 2-tailed *p* < 0.05.

## 3. Results

Among the patients considered, 175 were female and 220 were male; the mean age was 70.6 ± 11 years; the mean BMI was 27.8 ± 5.6 kg/m^2^. Of them 54.1% (214 patients) had permanent/persistent AF and 45.9% (181 patients) had paroxysmal AF.

The population was divided into three subgroups (terziles) depending on BMI value:

- First tertile: patients with BMI less than 25.3 kg/m^2^ (*n* = 127).
- Second tertile: patients with BMI between 25.3 kg/m^2^ and 29.3 kg/m^2^ (*n* = 137).
- Third tertile: patients with BMI greater than 29.3 kg/m^2^ (*n* = 131).

No statistically significant differences were found between the three tertiles regarding sex, age, systolic blood pressure and heart rate. However, a statistically significant difference was highlighted regarding the diastolic blood pressure value which was higher in the third tertile (*p* < 0.001). The CHA_2_DS_2_-VASc score was not statistically significantly different between the three groups. As regards the echocardiographic functional parameters, the following was noted: in the population examined LVMi increases progressively from the first to the third tertile (*p* = 0.001); however, a similar trend is not observed for the indexed left atrial volume, the ejection fraction and the Global Longitudinal Strain of the left ventricle and the E/E’ ratio; in fact, these values were not statistically significantly different between the three groups. As regards Peak Atrial Longitudinal Strain, its value was lower in the third tertile (14.2 ± 8.3%) compared to both the first (19 ± 11.5%) and the second tertile (17.8 ± 10.6%) in a statistically significant manner (*p* < 0.02) (Table 1).

**Figure 3:**
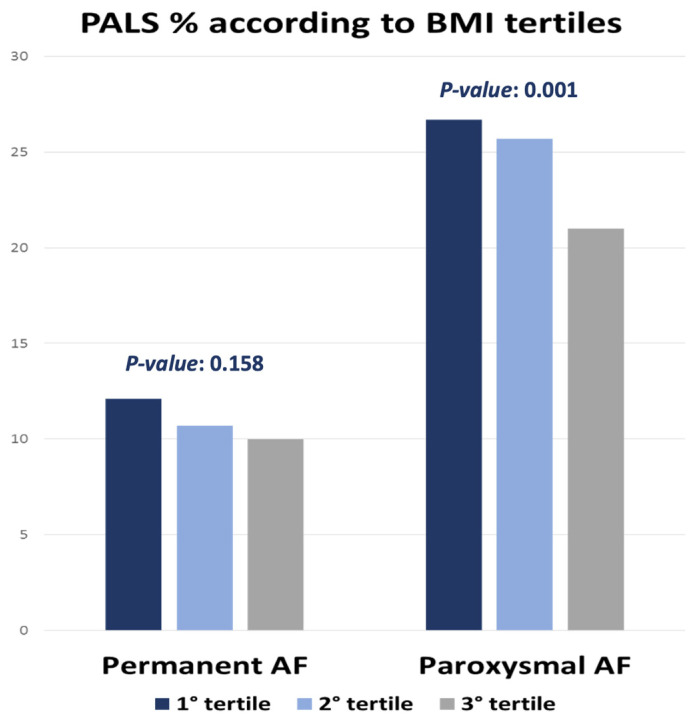
PALS reduction in a subanalysis based on the type of AF (permanent vs. paroxysmal). Abbreviations: PALS Peak Atrial Longitudinal Strain; BMI body mass index; AF atrial fibrillation.

However, in a subanalysis based on the type of AF, we found that in reality this reduction in PALS as BMI increases can be observed in the first and third tertiles (*p* = 0.001) of the group of paroxysmal fibrillating patients while this difference however, it was not statistically significant in patients with persistent/permanent AF (*p* = 0.158) as shown in Figure 3.

**Figure 4:**
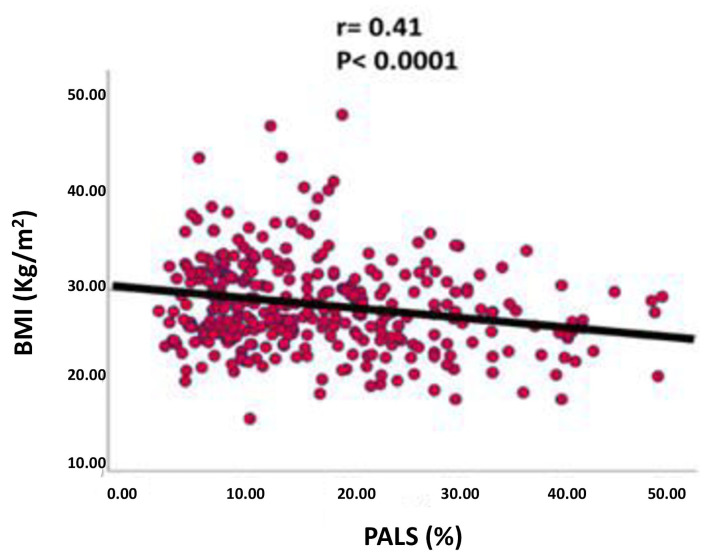
Relation between Body Mass index and Peak Atrial Longitudinal Strain by multilinear regression analysis.

Furthermore, in the population under examination, PALS was associated in a statistically significant manner with BMI (*p* < 0.001) but also with age, heart rate, LVMi, left ventricular EF, GLS, E/E’ ratio and systolic pulmonary arterial pressure (PAPs). However, after having conducted a multilinear regression analysis, adjusting these data for the CHA_2_DS_2_-VASc score, the LVMi, the left ventricular EF, the E/E’ ratio and the PAPs, we found that the BMI remained significantly associated independent with the PALS (coefficientstandardized β = −0.127, *p* < 0.02; Cumulative R^2^ = 0.41, SEE = 0.8%, *p* < 0.0001) as shown in Table 2 and Figure 4.

## 4. Discussion

In our study we investigated the role of obesity, assessed by the anthropometric parameter of BMI, on left atrial function, assessed by baseline echocardiography and speckle tracking, in patients with non-valvular AF.

Risk factors associated with atrial fibrillation (including obesity) [40] are associated, in some studies, with the onset of AF in patients with normal atrial dimensions, presupposing the existence of etiopathological mechanisms that are not expressed in atrial dilatation [41], thus giving rise to the need for a rigorous evaluation of left atrial function. The new echocardiographic techniques and, in particular, speckle tracking echocardiography, allow greater accuracy in the functional evaluation of the left atrium [37].

The PALS, which constitutes a marker of the atrial reservoir function, representing the point of maximum positivity of the strain curve, correlates better than the PACS with the prognostic data [42,43].

In our population of patients with non-valvular AF, the gradual reduction in PALS found when examining tertiles with increasing BMI shows us how overweight and obesity could have a detrimental effect on left atrial function [44].

Furthermore, BMI correlates with PALS even if we analyze this trend in the absence of numerous confounding factors (including CHA_2_DS_2_-VASc score) which could have an impact especially on obese categories with a higher BMI.

Overweight and obesity could increase the risk of AF through multiple mechanisms, such as structural and electrical atrial remodeling, which contributes to the creation of a proarrhythmic substrate. Ectopic deposition of adipose tissue in the heart has been associated with the prevalence and severity of atrial fibrillation. Epicardial adipose tissue, in particular, appears to constitute an important arrhythmogenic substrate that could explain the increased risk of AF related to obesity [21]. Furthermore, the amount of epicardial adipose tissue is a predictor of AF persistence [45]. The mechanisms by which epicardial fat contributes to the onset and progression of AF are not fully understood [46]. The pathophysiological mechanisms involve the infiltration of adipose tissue, the profibrotic and proinflammatory paracrine effects exerted by epicardial fat, as well as oxidative stress [47]. In fact, adipocytes, under the impulses of both physiological and pathophysiological stimuli, are capable of producing over 50 cytokines, hormones and peptides, grouped under the definition of “adipokines”, which play a very important role in the regulation of energy homeostasis and inflammation [48]. In obese individuals, hypertrophic adipocytes produce a series of molecules such as resistin, leptin, interleukin-6 (IL-6) and tumor necrosis factor-*α* (TNF-α) which determine a switch towards the proinflammatory state [49]. Increased sympathetic tone related to the dense innervation of cardiac adipose tissue depots contiguous with the atrium and pulmonary veins may also play a role [50]. The phenomena listed certainly contribute to varying degrees to the myocardial dysfunction characteristic of these patients.

Even though various mechanisms may play a role in the pathogenesis of cardiac dysfunction in obesity, early detection of these myocardial abnormalities may be important in management of the disease.

## 5. Limitations of the Study and Future Prospects

Among the limitations of our study it must certainly be recognized that it is an observational, non-prospective study and secondly it is a monocentric, not multicentric study, despite the number of patients enrolled being 395. The study also offers various food for thought; in fact, it could be interesting to investigate the relationship between atrial function indices analyzed by speckle tracking echocardiography and the risk of recurrence of atrial fibrillation not only in patients with obesity but also in other clinical settings, with the aim of carrying out an accurate evaluation of the patients’ risk profile for optimal therapeutic management.

## 6. Conclusions

In patients with non-valvular AF, overweight and obesity have a deleterious effect on left atrial function. This is demonstrated by the gradual reduction of PALS with increasing BMI across the various tertiles. BMI remains independently associated with PALS even after correction for various confounding factors including the CHA_2_DS_2_-VASc score. Therefore, BMI could be considered an additional factor in assessing cardiovascular risk in patients with non-valvular atrial fibrillation, in addition to the well-known CHA_2_DS_2_-VASc score.

## Figures and Tables

**Table 1:** Anthropometric, clinical and echocardiographic characteristics of patients with non-valvular atrial fibrillation divided into three tertiles based on body mass index values.

Variables	I Tertile (BMI < 25.3) N = 127	II Tertile (25.3 ≤ BMI ≤ 29.3) N = 137	III Tertile (BMI > 29.3) N = 131	Pa	Pb	Pc
Age (years)	71.9 ± 11.4	69.8 ± 11.5	70.1 ± 10.0	NS	NS	NS
SBP (mmHg)	127 ± 18	130 ± 17	129 ± 21	NS	NS	NS
DBP (mmHg)	74 ± 10	78 ± 10	77 ± 11	**<0.01**	NS	**<0.001**
BMI (kg/m^2^)	22.6 ± 2.1	27.1 ± 1.2	33.0 ± 3.6	**<0.0001**	**<0.0001**	**<0.0001**
CHA_2_DS_2_-VASc	3.2 ± 1.6	3.3 ± 1.7	3.4 ± 1.7	NS	NS	NS
LV Mass Index	42.0 ± 13.9	45.7 ± 15.5	49.0 ± 13.7	NS	NS	**0.001**
LV EF (%)	55.1 ± 10.3	57.2 ± 8.9	55.7 ± 8.7	NS	NS	NS
LV GLS (%)	18.7 ± 5.7	18.9 ± 4.7	18.5 ± 5.1	NS	NS	NS
E/e’	11.6 ± 5.5	11.9 ± 7.1	18.5 ± 5.1	NS	NS	NS
LA volume index	45.0 ± 16.1	43.9 ± 14.3	47.0 ± 14.3	NS	NS	NS
PALS (%)	19.0 ± 11.5	17.8 ± 10.6	14.2 ± 8.3	NS	**0.03**	**0.02**

Abbreviations: SBP: Systolic Blood Pressure; DBP: Diastolic Blood Pressure; BMI: Body Mass Index; LV Mass Index: left ventricular mass indexed by height ^2,7^; EF: Ejection Fraction; GLS: Global Longitudinal Strain; E/e’: ratio of transmitral pattern; LA volume index: left atrial volume indexed by BSA (body surface area); PALS: Peak Atrial Longitudinal Strain; Pa: *p*-value of I tertile compared to III ttertile; Pb: *p*-value of II tertile compared to III tertile; Pc: *p*-value of III tertile compared to I tertile; NS: not statistically significant.

**Table 2:** Multilinear regression analysis of variables with main prognostic value.

Dependent Variable	Determinants	β Coefficient	*p*-Value
PALS	CHA_2_DS_2_-VASc	−0.180	<0.001
	LV mass index	−0.112	0.04
	LV EF	0.228	<0.0001
	E/e’	−0.141	<0.01
	PAPs	−0.153	0.006
	BMI	−0.140	0.004

Abbreviations: PALS Peak Atrial Longitudinal Strain, LV left ventricular, EF Ejection Fraction, E/e’ ratio of transmitral pattern, PAPs systolic pulmonary arterial pressure, BMI Body Mass Index.

## Data Availability

Data presented in this study are available on request due to privacy and ethical restrictions.
